# Supplementary material for: Genome-Wide Transcriptome Profiling Reveals the Mechanisms Underlying Hepatic Metabolism under Different Raising Systems in Yak
Source: Animals (Basel). 2024 Feb 23;14(5):695. doi: 10.3390/ani14050695 (PMC10930694; doi:10.3390/ani14050695)
Supplement: Supplementary file 1 [file animals-14-00695-s001.zip › Supplementary Materials Table S1-6.docx]

Table S1 Basic diet composition of fattening group

| Item | Content(%) |
| --- | --- |
| Corn | 48.55 |
| Wheat Bran | 6.22 |
| Rapeseed Cake | 7.25 |
| Baking Soda | 0.25 |
| Dairy Salt | 0.25 |
| 4% Premix Compound | 1.5 |
| Oat Hay | 22 |
| Alfalfa Hays | 14 |
| Total | 100 |

Table S2 The growth performance of experimental yaks

| Item | Control | Test | P value |
| --- | --- | --- | --- |
| Initial weight (kg) | 215±22.72 | 244.50±28.03 | 0.049 |
| First month weight (kg) | 221.83±23.45 | 274.00±26.92 | 0.002 |
| Second month weight (kg) | 264.33±31.78 | 322.83±24.64 | 0.002 |
| Third month weight (kg) | 286±36.83 | 360.83±2.32 | 0.000 |
| Fourth month weight (kg) | 306.83±34.34 | 377.17±15.77 | 0.000 |
| End weight (kg) | 287.50±29.25 | 381.00±21.30 | 0.000 |
| Gain weight in the first month (kg) | 6.83±4.22 | 29.50±10.82 | 0.000 |
| Gain weight in the second month (kg) | 42.5±11.1 | 48.83±14.72 | 0.375 |
| Gain weight in the third month (kg) | 21.67±14.99 | 38.00±26.21 | 0.459 |
| Gain weight in the fourth month (kg) | 20.83±6.88 | 16.33±17.28 | 0.528 |
| Gain weight in the fifth month (kg) | -19.33±13 | 3.83±19.17 | 0.020 |
| Average daily gain (ADG) (kg/d) | 0.47±0.74 | 0.89±0.57 | 0.265 |
| Total weight gain (TWG) (kg) | 72.50±13.49 | 136.50±30.39 | 0.000 |

Table S3 Information of primers used in validation

| Primer name | Primer sequence (5’→3’) |
| --- | --- |
| LAMA2-F | TCGCTCACAAAGACCCAAGA |
| LAMA2-R | AATACACACACCCCCTCCAA |
| IGF1-F | CCATCACATCCTCCTCGCAT |
| IGF1-R | CTGCACTCCCTCTACTTGTGT |
| APOA1-F | TCTGGAAAAGGAGACCGCGT |
| APOA1-R | TCACTGGGCGTTCAGCTTCT |
| FABP1-F | TCAAGGGGGTGTCGGAAATC |
| FABP1-R | TCGCCCTTCGTCATGGTACT |
| SLC27A5-F | CCTCAGTTGCCTGGACCTTG |
| SLC27A5-R | CTCGAAGGGGGACAGCATTC |
| CPT1B-F | CAACCAACTACGTGAGCGACT |
| CPT1B-R | AAAATCGGCCCTTGTGGTAG |
| HMGCS2-F | AGGGCTTAGAGGAACCCACAT |
| HMGCS2-R | TGTCTTCCAGCTTTAGTCCCC |
| PLIN5-F | GTGTGGAGCTGAAGCGATCT |
| PLIN5-R | GGCATGGTGTTTCTTCTGCC |

Table S4 Results of GO functional significant enrichment analysis of differentially expressed gene

| Category | GOID | Description | Gene number |
| --- | --- | --- | --- |
| BP | GO:0055114 | oxidation-reduction process | 80 |
| CC | GO:0005581 | collagen trimer | 7 |
| CC | GO:0005576 | extracellular region | 35 |
| MF | GO:0016491 | oxidoreductase activity | 87 |
| MF | GO:0048037 | cofactor binding | 55 |
| MF | GO:0046906 | tetrapyrrole binding | 28 |
| MF | GO:0016705 | oxidoreductase activity, acting on paired donors, with incorporation or reduction of molecular oxygen | 27 |
| MF | GO:0020037 | heme binding | 26 |
| MF | GO:0005506 | iron ion binding | 26 |
| MF | GO:0005201 | extracellular matrix structural constituent | 7 |
| MF | GO:0004497 | monooxygenase activity | 7 |
| MF | GO:0050662 | coenzyme binding | 22 |
| MF | GO:0016746 | transferase activity, transferring acyl groups | 17 |

Table S5 Candidate KEGG pathways and genes

| KEGG Pathway | Up-regulated gene | Down-regulated gene |
| --- | --- | --- |
| PPAR signaling pathway | APOA1/FABP1 | \| EHHADH/FADS2/SLC27A5/ACADM/CPT1B/ACOX2/HMGCS2/PLIN5/ACAA1 \| \| --- \| |
| PI3K-Art | IGF1/LAMA2 | FGFR4 |
| Fatty acid degradation | / | ACAA1/EHHADH/ALDH9A1/ACADM/CPT1B/ECHS1 |
| ECM-receptor interaction | LAMA2 | / |

Table S6 Comparison of meat quality between the feeding group and the grazing group

| Item | Fattening group | Grazing group |
| --- | --- | --- |
| PH_45min_ value | 6.33±0.24^A^ | 6.88±0.33^B^ |
| Lightness L^*^_45min_ | 6.98±1.19 | 6.32±1.05 |
| Redness a^*^_45min_ | 29.29±2.57 | 28.12±3.21 |
| Yellowness b^*^_45min_ | 6.42±1.45 | 5.67±1.41 |
| PH_24h_value | 5.70±1.43 | 5.49±0.26 |
| Lightness L^*^_24h_ | 8.03±0.79^a^ | 8.86±1.38^b^ |
| Redness a^*^_24h_ | 32.68±1.37 | 33.23±1.34 |
| Yellowness b^*^_24h_ | 8.62±1.09^a^ | 9.64±1.14^b^ |
| Cooked meat percentage/% | 72.79±1.70^A^ | 82.18±3.84^B^ |
| Water loss rate/% | 17.37±4.02 | 15.08±2.66 |
| Shear force/% | 14.89±2.59^A^ | 17.75±1.90^B^ |
