# Supplementary figures and images for: Genome-Wide Transcriptome Profiling Reveals the Mechanisms Underlying Hepatic Metabolism under Different Raising Systems in Yak
Source: Animals (Basel). 2024 Feb 23;14(5):695. doi: 10.3390/ani14050695 (PMC10930694; doi:10.3390/ani14050695)

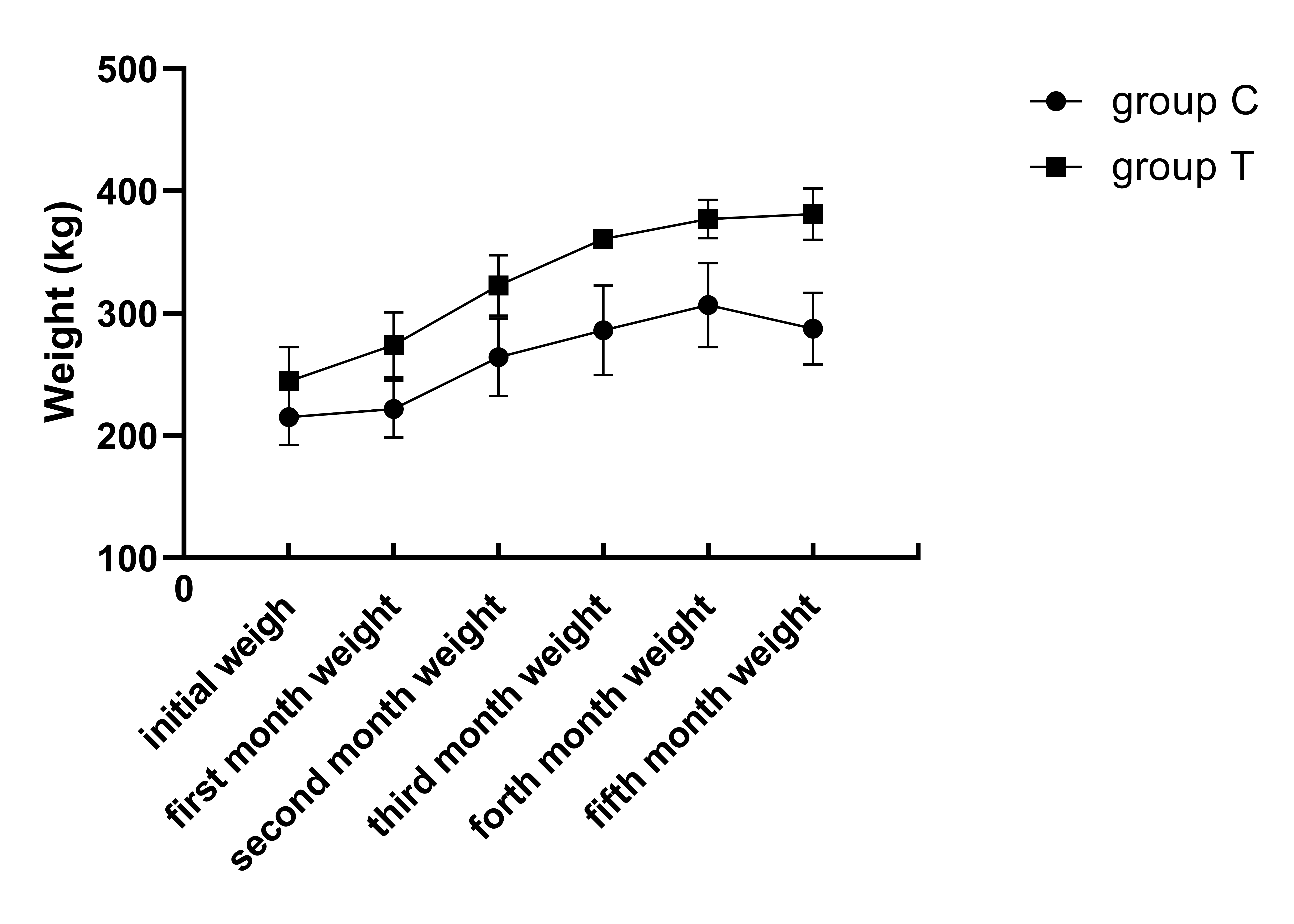

Supplement: Supplementary file 1 [file animals-14-00695-s001.zip › Supplementary Materials Figure S1 .tif]

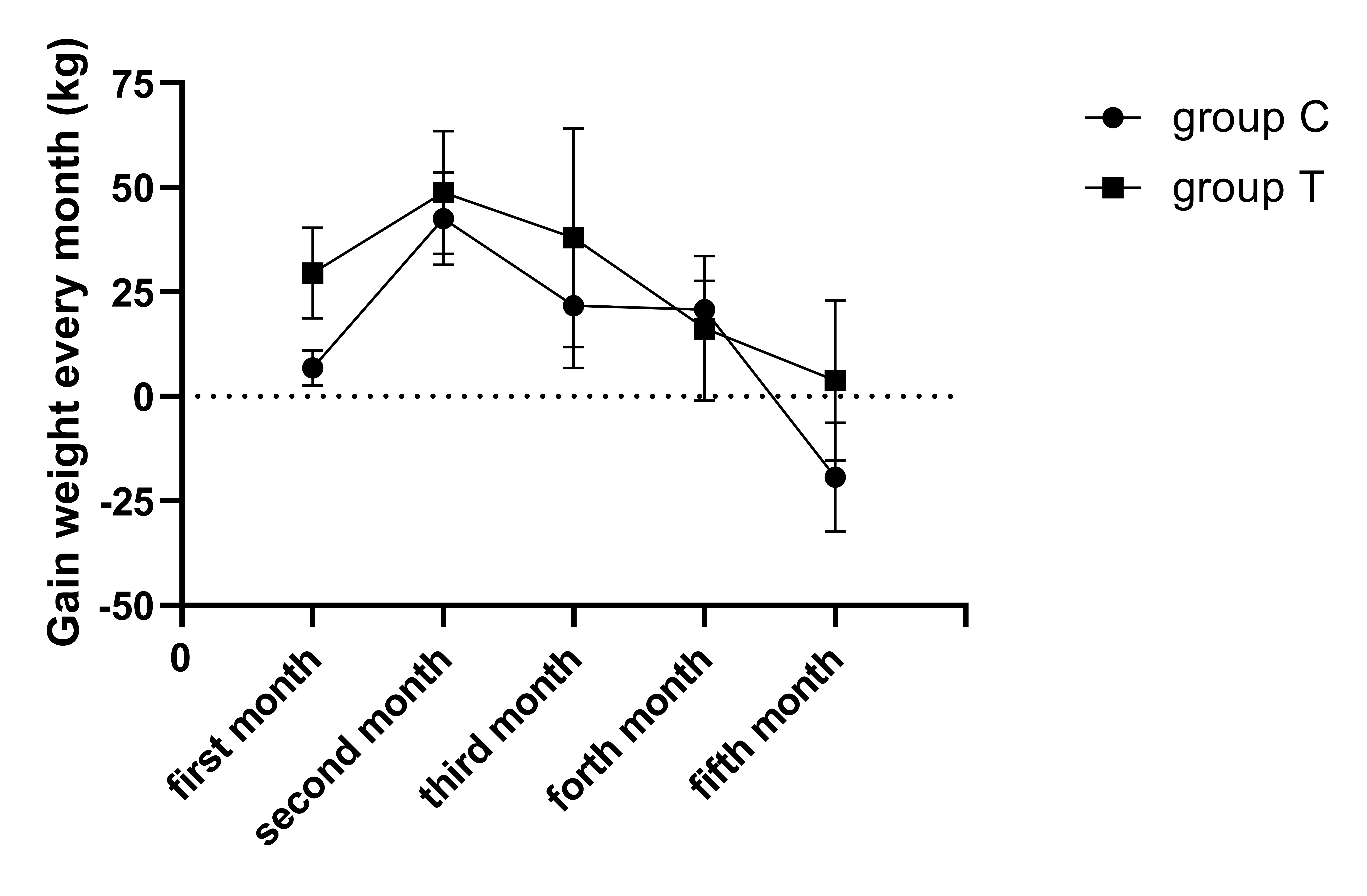

Supplement: Supplementary file 1 [file animals-14-00695-s001.zip › Supplementary Materials Figure S2 .tif]

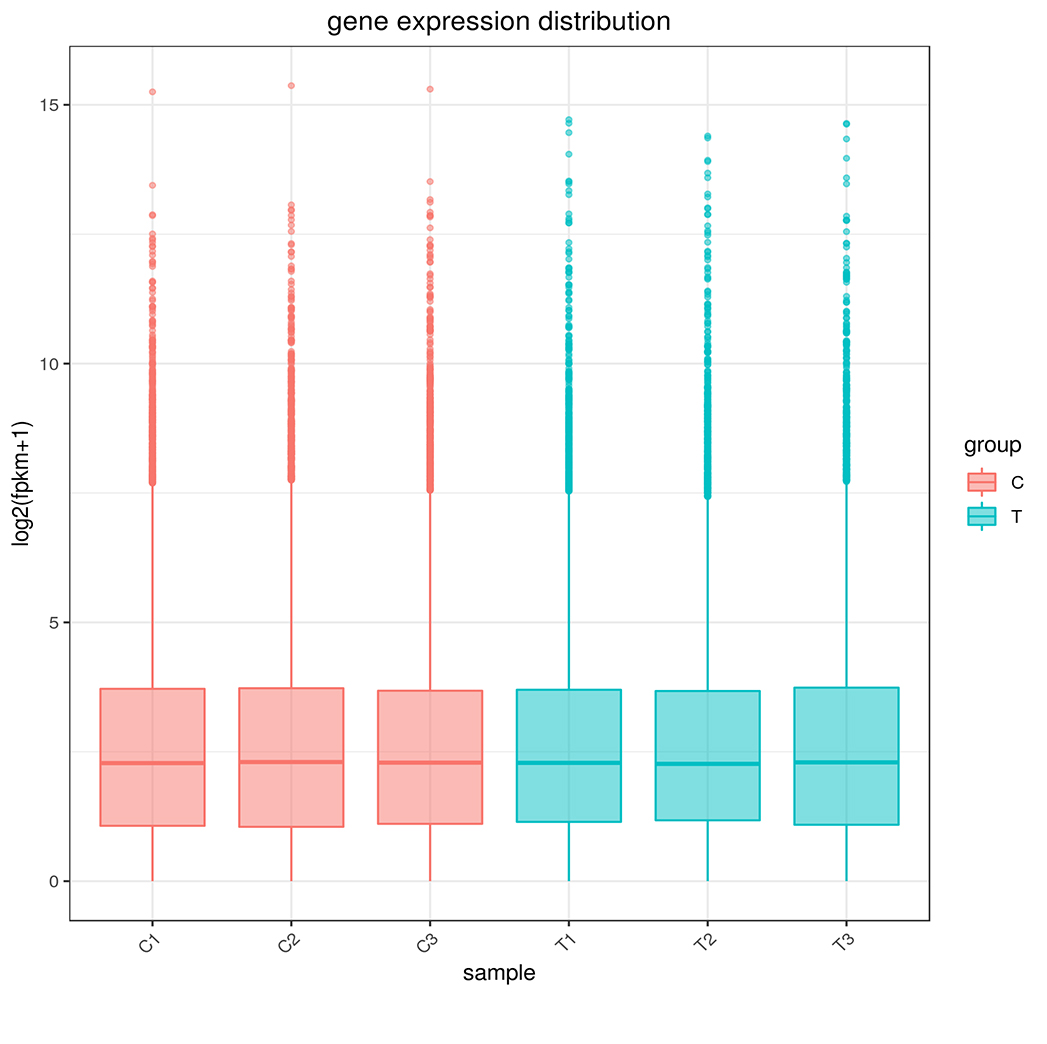

Supplement: Supplementary file 1 [file animals-14-00695-s001.zip › Supplementary Materials Figure S3 .jpg]

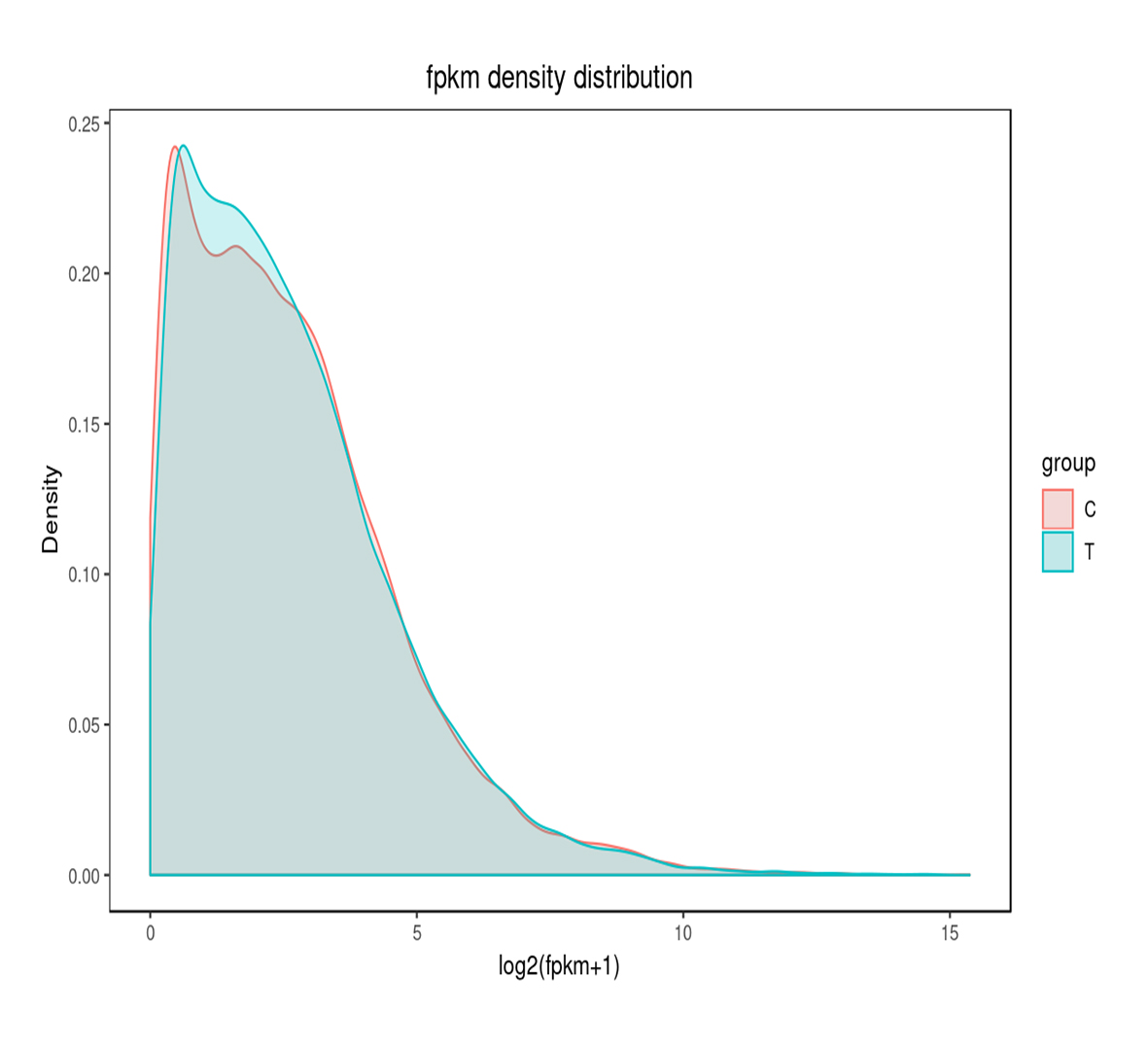

Supplement: Supplementary file 1 [file animals-14-00695-s001.zip › Supplementary Materials Figure S4 .jpg]

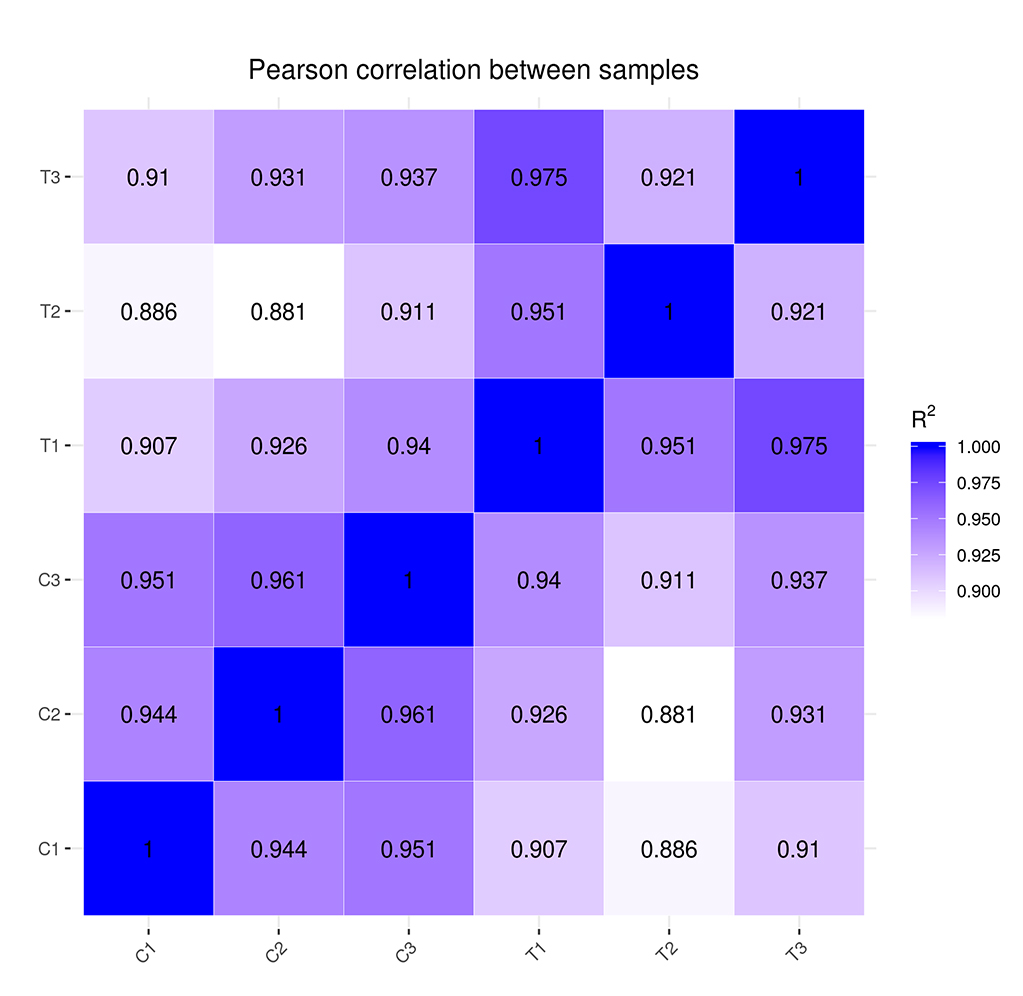

Supplement: Supplementary file 1 [file animals-14-00695-s001.zip › Supplementary Materials Figure S5 .jpg]

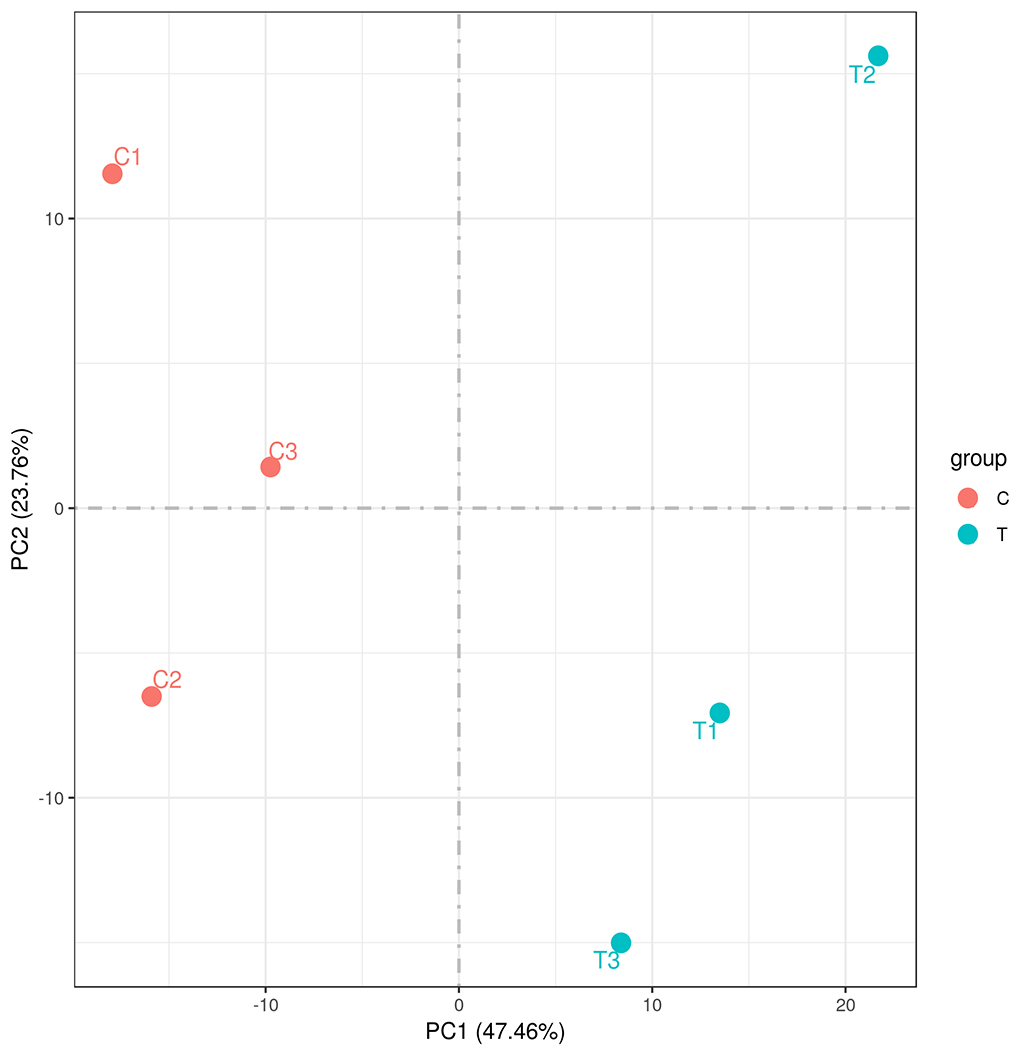

Supplement: Supplementary file 1 [file animals-14-00695-s001.zip › Supplementary Materials Figure S6 .jpg]
